# Gender differences in Korean adolescents who died by suicide based on teacher reports

**DOI:** 10.1186/s13034-019-0274-3

**Published:** 2019-03-11

**Authors:** Song Jung, Dayoung Lee, Sungjun Park, Kangwoo Lee, Yong-Sil Kweon, Eun-Jin Lee, Kyung Hee Yoon, Hannah Cho, Hyeji Jung, Ah Reum Kim, Bo-Ram Shin, Hyun Ju Hong

**Affiliations:** 1 Department of Psychiatry, Hallym University Sacred Heart Hospital, 22, Gwanpyeong-ro 170beon-gil, Dongan-gu, Anyang-si, Gyeonggi-do South Korea; 2 Hallym University Suicide and School Mental Health Institute, Hallym University Sacred Heart Hospital, 176-14, Gwanpyeong-ro, Dongan-gu, Anyang-si, Gyeonggi-do South Korea; 3 Department of Psychiatry, Uijeongbu St. Mary’s Hospital, College of Medicine, The Catholic University of Korea, Seoul, South Korea; 4 Department of Social Welfare, Suwon Science College, 288, Seja-ro, Jeongnam-myeon, Hwaseong-si, Gyeonggi-do South Korea; 5 Department of Child Welfare and Studies, Sookmyung Women’s University, 100 Cheongpa-ro 47-gil, Yongsan-gu, Seoul, South Korea; 6 Department of Social Studies Education, Incheon National University, Songdo-dong, 119 Academy-ro, Yeonsu-gu, Incheon, South Korea

**Keywords:** Suicide, Adolescent, Gender

## Abstract

**Background:**

We investigated the characteristics of adolescents who committed suicide in South Korea, and how these characteristics differed by gender.

**Method:**

Data from middle and high school students who committed suicide between 2014 and 2016 were analyzed. We evaluated differences in suicide method and place, personal characteristics, and school life characteristics by gender using the Chi square test and *t* test.

**Results:**

Jumping from a high place was the most common suicide method for both male and female students. A significantly greater proportion of female adolescents had experienced depressive symptoms, previous self-injury, previous suicide attempts, and had problems with school attendance and peers. Additionally, they were more likely to be classified as high risk according to a school-based mental health screening test and to utilize professional mental health treatment services.

**Conclusion:**

Our results demonstrate that adolescents who committed suicide exhibited gender differences in personal characteristics and school life. These characteristics might aid in the development of adolescent suicide policies and intervention programs.

**Electronic supplementary material:**

The online version of this article (10.1186/s13034-019-0274-3) contains supplementary material, which is available to authorized users.

## Background

Over the past two decades, suicide rates among adolescents (individuals aged 15–19 years old) in Organisation for Economic Co-Operation and Development (OECD) countries declined noticeably from 8.3 per 100,000 teenagers in 1990 to 6.4 in 2013 [1]. Nevertheless, suicide remained the second leading cause of death of youth in 2014 [2], suggesting that it still needs constant attention and a solution.

Suicidal behavior in adolescents is associated with a range of factors, including psychiatric disorders, alcohol and substance abuse, previous suicide attempt(s), family history of suicide or mental disorders, and low family support [3–6]. However, it can be difficult to clearly identify the characteristics of adolescent suicides because actual completed suicides are relatively rare among adolescents compared to among adults; in addition, most studies focused on either suicide ideation or suicide attempts [7]. Some studies have suggested that there is a gender difference in suicide [4, 8, 9]. In general, suicide ideation and attempts are more common among females than among males, but the suicide mortality rate is higher among males. This feature is referred to as the “gender paradox” in suicide [7, 10, 11]. The most common explanation for the gender paradox is that males are more likely to choose lethal suicide methods than are females. The gender difference in suicide also appears to vary among countries and cultures, and even within a single country. Age is also a known influencing factor of the gender difference [4, 7, 11–13].

Adolescents experience numerous psychological and social changes. During this critical period, most students attend school for much of their day, nearly every day [14]. Several studies have demonstrated that school-based interventions are effective in reducing suicide attempts and suicide ideation in adolescents [15, 16]. Teachers interact directly with potentially risky students and can act as gatekeepers in suicide prevention by recognizing suicide warning signs and identifying changes in their students [17]. However, there is little research on teachers’ perspectives on suicide among adolescents [18]. Therefore, we investigated the characteristics of Korean adolescents who committed suicide, and explored the gender differences in these characteristics, based on teacher-written student suicide reports.

## Methods

### Data collection

This study focused on middle and high school students who committed suicide between 2014 and 2016. The number of suicides per year was 89 in 2014, 90 in 2015, and 105 in 2016. The study data were obtained from the Ministry of Education, which has been collecting student suicide data since 2015. When an elementary, middle, or high school student dies by suicide, their teacher must submit a “student suicide report” to the education office within 7 days. We also collected data on suicides that occurred before 2015 from available suicide cases. The student suicide report contains a variety of data, including demographic characteristics, suicide-related information (e.g., method, place, suicide note), personal traits, family environment, physical and mental health, history of suicide attempts, school life, history of school-based mental health support, etc. The items use a variety of answer formats, including multiple-choice, single-choice, or open-ended. Details of the contents of the report are shown in Additional file 1: Table S1.

Since 2016, the report has also included items on linguistic, behavioral, and emotional changes prior to suicide attempts have been added. Linguistic signs are verbal expression of death, suicide, suicide method, physical discomfort, longing for the afterlife or mention someone who died by suicide. Behavioral signs are changes of sleep or appetite, planning of suicide, self-mutilating behaviors, indifference to appearance management, substance abuse, avoidant of interpersonal relationship, inattention or action to finish personal life. Emotional signs are depressive mood, irritability, hopelessness, despair, loneliness, guilty feeling or loss of interests. Therefore, these items were analyzed only for the year 2016. The study was approved by the Institutional Review Board of Hallym University Sacred Heart Hospital.

### Data analysis

We used the Chi square test, Fisher’s exact test, and *t*-test to assess gender differences in characteristics of adolescents who died by suicide. The significance level of the statistical tests was set to 0.05. All data analyses were performed using SPSS Statistics 23.0.

## Results

### Demographics

**Fig. 1:**
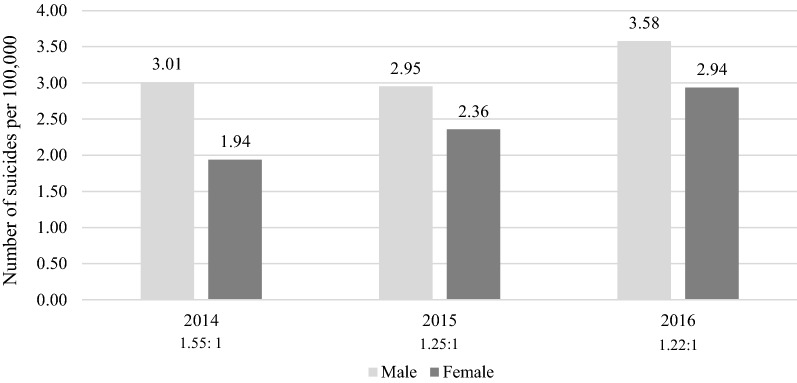
Suicide rate by gender from 2014 to 2016. The gender ratio (male:female) is presented below each year

Of the 284 adolescents who died by suicide between 2014 and 2016, 168 were male (59.2%) and 116 were female (40.8%). As shown in Fig. 1, the suicide rate of males was higher than that of females. The gender ratio (male: female) in suicide rates was 1.55:1, 1.25:1 and 1.22:1 from 2014 to 2016 respectively. The mean (*SD*) age at death was 15.98 (1.47) years for males and 15.75 (1.45) years for females.

### Method of suicide

The most common method of suicide in both male and female students was jumping from a high place (male 71.0%, female 70.8%), followed by hanging (male 25.0%, female 22.1%). Among male students, 1.2% and 1.9% committed suicide by gas inhalation and poisoning, respectively; females, by contrast, favored gas inhalation (6.2%) over poisoning (0.9%; Table 1). None of these gender differences were statistically significant.

**Table 1:** Method and place of suicide by gender

	Male		Female		Total	
	n	%	n	%	n	%
*Method of suicide*						
Jumping from high place	115	71.0	80	70.8	195	70.9
Hanging	42	25.9	25	22.1	67	24.4
Poisoning	2	1.2	1	0.9	3	1.1
Gas inhalation	3	1.9	7	6.2	10	3.6
Total	162	100.0	113	100.0	275	100.0
*Place of suicide*						
Residence	85	50.9	61	52.6	146	51.6
Other than residence	82	49.1	55	47.4	137	4.8
Total	167	100.0	116	100.0	283	100.0

### Place of suicide

Place of suicide was classified as residence (own home, friend’s home, relative’s home) and other than residence (bridge, park, school, hospital, or another building). Among males, roughly half committed suicide in a residence or in a place other than a residence (50.9% vs. 49.1%). In contrast, slightly more females (52.6%) committed suicide in a residence than in a place other than a residence (47.4%; Table 1). The differences between the genders were not statistically significant.

### Personal and family characteristics

Among the family and personal characteristics, economic status, depressive symptoms, previous self-injury, and previous suicide attempt(s) significantly differed by gender (Table 2). Family economic status was significantly lower among female adolescents (*p *= 0.034), while depressive symptoms (*p *= 0.090), history of previous self-injury (*p *= 0.012), and history of suicide attempts (*p *= 0.043) were more common. Among male adolescents, 3.6% and 5.7% had experienced self-injury and a suicide attempt within 1 year prior to the suicide, respectively. In contrast, 19.5% of female adolescents attempted self-injury and 19.4% attempted suicide (roughly 4 times the rates in males).

**Table 2:** Personal characteristics of adolescents who committed suicide by gender

	Male		Female		χ^2^
	N	%	N	%	
*Parents*					
Living together	74	67.9	120	75.0	ns
Separation or divorce	29	26.6	31	19.4	
Dead (one or both)	6	5.5	9	5.6	
*Economic status*					
High	17	10.5	11	9.9	6.789*
Middle	117	72.2	66	59.5	
Low	28	17.3	34	30.6	
*Personality*					
Introvert	111	75.0	64	66.7	ns
Extrovert	37	25.0	32	33.3	
*Depressive symptoms*					
Yes	37	22.7	36	31.9	2.878^†^
No	126	77.3	77	68.1	
*Impulsivity*					
Yes	134	81.2	95	83.3	ns
No	31	18.8	19	16.7	
*Smoking*					
Yes	25	15.2	10	8.8	NS
No	139	84.8	103	91.2	
*Drinking*					
Yes	16	9.8	8	7.1	NS
No	148	90.2	105	92.9	
*Previous self*-*injury*					
Yes	2	3.6	8	19.5	6.344*
No	53	96.4	33	80.5	
*Previous suicide attempt*					
Yes	3	5.7	7	19.4	4.084*
No	50	94.3	29	80.6	

^†^*p *< 0.10, *p < 0.05, **p < 0.01, ***p < 0.001

### School Life Characteristics

As shown in Table 3, gender differences in the school life characteristics of adolescents who committed suicide were also identified. Male adolescents showed significantly better school attendance than did female adolescents in terms of rates of tardiness, leaving early, or absence (*p *= 0.000). Among males, unauthorized absences were more common than were absences due to illness (13.2% vs. 6.0%), while in female adolescents, illness-related absences were more common than were unauthorized ones (22.1% vs. 5.3%).

**Table 3:** School life characteristics of adolescents who committed suicide by gender

	Male		Female		χ^2^
	N	%	N	%	
*Academic achievement*					
Above average	30	18.2	23	20.7	ns
Average	80	48.5	45	40.5	
Below average	55	33.3	43	38.7	
*School attendance*					
Good	135	80.8	80	70.8	19.137***
Tardiness/leave early/absence (due to illness)	10	6.0	25	22.1	
Tardiness/leave early/absence (without notice)	22	13.2	6	5.3	
*Friendship*					
Amicable	147	89.1	89	81.7	3.041^†^
Discord/isolation	18	10.9	20	18.3	
*School adjustment problems*					
Yes	10	6.1	5	4.4	ns
No	154	93.9	108	95.6	
*Perpetrator of school violence*					
Yes	6	3.7	6	5.3	ns
No	158	96.3	107	94.7	
*School*-*based mental health screening test*^*a*^					
Normal group	126	84.6	69	73.4	4.528*
High-risk group	23	15.4	25	26.6	
*Counseling sessions in school* ^*b*^					
Yes	26	20.2	23	26.1	ns
No	103	79.8	65	73.9	
*Professional help* ^*c*^					
Yes	20	15.5	25	28.4	5.301*
No	109	84.5	63	71.6	
*Warning signs* ^*d*^					
Yes	11	17.5	9	20.0	6.756*
No	49	77.8	27	60.0	
Unknown	3	4.8	9	20.0	

^†^*p *< 0.10, *p < 0.05, **p < 0.01, ***p < 0.001

^a^Students classified as the high-risk group requiring priority management and intervention in nationwide school-based mental health screening test

^b,c^Students who used school counseling or external resources (counseling center or mental hospital) before suicide

^d^Students who showed pre-suicide linguistic, behavioral, or emotional changes according to teachers

Problems such as isolation and discord in friendships were more common in female adolescents than in male adolescents. Among male adolescents, about 90% had good friendships and 10.9% had problems in their friendships; in contrast, these rates were 81.7% and 18.3% among females, respectively.

Students were categorized into normal or high-risk groups according to their results on the nationwide school-based mental health screening test conducted at their schools. Female adolescents who committed suicide were more commonly classified as high-risk (i.e., requiring priority management and intervention) compared to their male counterparts (*p *= 0.033). Among male and female adolescents, 84.6% and 73.4% were classified as normal, respectively, while 15.4% and 26.6% were classified as high-risk. There were no statistically significant gender differences in the rates of using school counseling before the suicide (male = 20.2%; female = 26.1%, *p *= 0.301), but a significantly greater proportion of females than males had experienced professional treatment services (e.g., visiting an external counseling center or mental hospital; male = 15.5%; female = 28.4%, *p *= 0.021).

When focusing only on the 2016 data, there were statistically significant gender differences in pre-suicide linguistic, behavioral, or emotional changes in adolescents as perceived by teachers (*p *= 0.034). Among the adolescents who committed suicide in 2016, 17.5% of males and 20.0% of females showed such changes. Approximately 78% of male adolescents exhibited no pre-suicide changes, while for 4.8% the answer was not clear. These rates among female adolescents were 60% and 20%, respectively.

## Discussion

Using student suicide reports written by teachers, we investigated the characteristics of adolescents who committed suicide in South Korea between 2015 and 2016, and then explored the differences in these characteristics by gender. The methods and place of suicide were similar between male and female adolescents who committed suicide. However, a greater proportion of female adolescents than male adolescents experienced depressive symptoms, previous self-injury, and suicide attempts. Furthermore, a lower proportion of male adolescents experienced problems with school attendance and friendships, were classified as high-risk according to the school-based mental health screening test, and received professional help for their mental health.

The suicide rate of male adolescents was higher than that of female adolescents, but the gender ratio (male: female) in suicide rate was relatively lower than was that reported in Western countries. We also observed no gender difference in suicide method or place. Ahn et al. [12] reported that suicide method is a major determinant of the difference in gender ratio of suicide rate among countries. The lethality of the suicide method chosen by a person with an intention to commit suicide is related to the actual suicide rate. Jumping from a great height is one of the more violent methods, and was the most common suicide method among both male and female adolescents in South Korea. Accordingly, the low gender ratio of the suicide rate can be explained in terms of the similarity of suicide methods between male and female adolescents.

A greater proportion of female adolescents had depressive symptoms, history of self-injury, and history of suicide attempts. These results are consistent with the findings of previous studies reporting that suicide is strongly related to depression [19], and that the rates of self-harm and suicide attempts are much higher in female adolescents than in male adolescents [4, 9, 20]. Previous experience of self-harm and suicide attempts are also known to be the most important risk factors of suicide completion [21]. Thus, the relatively high rate of these factors in female adolescents might indicate that they have a greater potential risk of suicide.

As for the school life characteristics, a greater proportion of male adolescents who committed suicide had better school attendance than did female adolescents, while a lower proportion had problems with their friendship. Adolescence is, in general, a period in which individuals’ main source of attachment shifts from their parent to their peers; thus, the influence of peer relationships is strengthened [22]. Bearman and Moody [23] revealed that social isolation and disconnected friendships significantly increased suicidal ideation among female adolescents but not male adolescents. Winterrowd et al. [24] also showed that the relationship between friendship problems and suicidality differed by gender and ethnicity. These findings suggest that adolescent suicidality might be influenced by poor quality of friendship, such as isolation from peers, and that these effects vary by gender.

Mental health problems are a major risk factor of suicide among both male and female adolescents; however, the strength of the association between mental health problems and suicide might differ by gender. We found that 15% of male adolescents and 27% of female adolescents who committed suicide were classified as high-risk according to a school-based mental health screening test. Given the fact that 3–5% of regular students are screened as high-risk in 2014–2016, the test appears to contribute to early detection of students with suicide risk. We also identified gender differences in the use of professional mental health services: The ratio of female adolescents who used counseling centers or mental hospitals before suicide was about twice that of male adolescents. Several previous studies have shown that males who completed suicides were less likely to have received mental health services than were females [25, 26]. These gender differences might be due to the fact that males are less likely to seek help for mental health problems, and are more likely to seek out alternative solutions than professional help [27]. In other words, many adolescents who commit suicide do not appear to receive sufficient help beforehand. In the case of male adolescents, it might be more difficult for a school or teacher to identify high-risk suicide groups and manage them.

Finally, the patterns of pre-suicide linguistic, behavioral, and emotional changes in adolescents as perceived by teachers differed by gender. Although male and female adolescents showed similar rates of reporting such changes, the rate of reporting a lack of clarity about whether the changes had occurred or not among females was about four times the rate among males. This might be attributed to the influence of various factors, such as the characteristics of the teacher and student or their interactions.

This study has some limitations. First, the student suicide report might not accurately capture students’ information because it needs to be submitted within 1 week of the suicide case. Particularly, personal information such as past diagnosed or treated psychiatric illnesses or a family history of suicide cannot be accessed by teachers. Moreover, some items in the report might be influenced by teachers’ subjective evaluation.

Although gender differences are often reported in suicide research, most past studies on adolescents focused on those who attempted suicide or have suicidal ideation, rather than students who have completed suicide. The present study provides greater understanding of the gender differences in adolescents who committed suicide using quantitative data. In addition, various aspects of the school life of students who died by suicide, including school-level mental health intervention service and pre-suicide signs perceived by teachers, were explored.

## Conclusion

Our findings demonstrated that adolescents who committed suicide exhibited many gender differences in terms of their personal and school life characteristics, although their suicide methods were similar. To enhance the effectiveness of school-based suicide prevention policies, teachers must understand the characteristics of the adolescents who die by suicide and be actively involved in school-based counselling programs. It might also be necessary to develop more elaborated policies (intervention programs) reflecting the gender difference.

## Additional file

**Additional file 1: Table S1.** Contents of student suicide case report.
