# Supplementary material for: Gender differences in Korean adolescents who died by suicide based on teacher reports
Source: Child Adolesc Psychiatry Ment Health. 2019 Mar 11;13:12. doi: 10.1186/s13034-019-0274-3 (PMC6410496; doi:10.1186/s13034-019-0274-3)
Supplement: Supplementary file 1 — Additional file 1: Table S1. Contents of student suicide case report. [file 13034_2019_274_MOESM1_ESM.docx]

Table S1. Contents of student suicide case report

| **Categories of questions** | **Types of questions** | **Contents of questions** |
| --- | --- | --- |
| basic information | multiple choice | student’s gender, birthday, educational stage, name of school, school district, school type |
| suicide related information | multiple choice or single answer | date of suicide, location of suicide, method of suicide, types of suicide note |
|  | descriptive | details of suicide incident, description of suicide note |
| Prevention and postvention of school | multiple choice or single answer | Prevention programs and postvention action of school |
|  | descriptive | School’s responses to media and bereaved family |
| student characteristics | descriptive | description about school life, peer relationship, family situation, psychological and behavioral characteristics of students |
| family information | multiple choice or single answer | student’s family type, members of cohabitant, residence type, family income, main earner of family, religion, parental occupations |
|  | 4 point rating | closeness with parents, personality perceived by teacher |
| school life | multiple choice or single answer | student’s academic achievement, school attendance, school/legal punishment, emotional and behavioral status perceived in school, health status, previous suicidal behavior, experience of loss or suicide, types of usual troubles |
|  | 4 point rating | Level of closeness with teacher, sense of belonging to school, |
| school based mental health screening test | multiple choice | test result, linkage to school and community facilities, result of intervention |
| identification and intervention of students’ mental problems of school | multiple choice | Identification of student’s problems apart from school-based mental health screening test, status of intervention, intervention facilities supporting students, result of intervention |
| main reasons of student's suicide | descriptive | description of teacher's opinion about student’s suicide |
| warning signs of suicide and peer relation | multiple choice or single answer | student’s linguistic, behavioral, emotional changes before suicide |
|  | descriptive | description about changes in any aspects of student life before suicide |
|  | 4 point rating | level of closeness with friends |
| emotional and behavioral status of students | rating scale | strengths and difficulties questionnaire (teacher) |
